# Fourier Transform Infrared Polarization Contrast Imaging Recognizes Proteins Degradation in Lungs upon Metastasis from Breast Cancer

**DOI:** 10.3390/cancers13020162

**Published:** 2021-01-06

**Authors:** Karolina Chrabaszcz, Katarzyna Kaminska, Cai Li Song, Junko Morikawa, Monika Kujdowicz, Ewelina Michalczyk, Marta Smeda, Marta Stojak, Agnieszka Jasztal, Sergei G. Kazarian, Kamilla Malek

**Affiliations:** 1 Faculty of Chemistry, Jagiellonian University, Gronostajowa 2 St., 30-387 Krakow, Poland; karolina.chrabaszcz@doctoral.uj.edu.pl (K.C.); katarzyna1.kaminska@uj.edu.pl (K.K.); monika.kujdowicz@uj.edu.pl (M.K.); ewelina.michalczyk95@gmail.com (E.M.); 2 Jagiellonian Centre for Experimental Therapeutics, Jagiellonian University, Bobrzynskiego 14 St., 30-384 Krakow, Poland; marta.wojewoda@jcet.eu (M.S.); marta.stojak@jcet.eu (M.S.); agnieszka.jasztal@jcet.eu (A.J.); 3 Department of Chemical Engineering, Imperial London College, South Kensington Campus, London SW72AZ, UK; cai.song13@imperial.ac.uk; 4 School of Materials and Chemical Technology, Tokyo Institute of Technology, Tokyo 152-8550, Japan; morikawa.j.aa@m.titech.ac.jp; 5 Department of Pathomorphology, Medical Faculty, Jagiellonian University Medical College, Grzegorzecka 16 St., 31-531 Krakow, Poland

**Keywords:** FTIR imaging, polarization contrast imaging, protein degradation, metastatic niche, micro-metastasis

## Abstract

**Simple Summary:**

Several lung extracellular matrix (ECM) proteins are involved in the formation of a metastatic niche in pulmonary metastasis and they accompany the cancer progression. Its gradual remodeling does not induce compositional changes of its components, but it is related to the re-distribution of individual proteins, their cross-linking and spatial arrangement within the tissue. The combination of FTIR and FTIR polarization contrast (PCI) imaging, as rapid, non-destructive, and label-free techniques, allows for the determination of protein alternations occurring in lungs that are affected by breast cancer metastasis. Both have the potential to characterize biochemical changes of the metastatic target, can determine phenotypes of tissue structures, and deliver a novel spectroscopic marker panel for the recognition of metastasis environment.

**Abstract:**

The current understanding of mechanisms underlying the formation of metastatic tumors has required multi-parametric methods. The tissue micro-environment in secondary organs is not easily evaluated due to complex interpretation with existing tools. Here, we demonstrate the detection of structural modifications in proteins using emerging Fourier Transform Infrared (FTIR) imaging combined with light polarization. We investigated lungs affected by breast cancer metastasis in the orthotopic murine model from the pre-metastatic phase, through early micro-metastasis, up to an advanced phase, in which solid tumors are developed in lung parenchyma. The two IR-light polarization techniques revealed, for the first time, the orientational ordering of proteins upon the progression of pulmonary metastasis of breast cancer. Their distribution was complemented by detailed histological examination. Polarized contrast imaging recognised tissue structures of lungs and showed deformations in protein scaffolds induced by inflammatory infiltration, fibrosis, and tumor growth. This effect was recognised by not only changes in absorbance of the spectral bands but also by the band shifts and the appearance of new signals. Therefore, we proposed this approach as a useful tool for evaluation of progressive and irreversible molecular changes that occur sequentially in the metastatic process.

## 1. Introduction

Lungs are the primary organ of the respiratory system and due to their strong blood supply and high oxygen availability they are one of the most common sites of metastasis from primary breast cancer. The cancer cells retained at a given stage of the cell cycle adhere to capillary beds, before penetration into the organ parenchyma and then proliferate and promote angiogenesis within a targeted organ [1]. Metastasis causes extensive changes in lung tissue and proteome, ranging from remodeling of the extracellular matrix (ECM) to fibrosis and formation of solid tumors. 

The most abundant proteins in the lungs are collagens, mostly type I, III, and IV [2]. Laminin, the main protein of basal membrane, and elastin, a protein occurring in organs requiring elasticity, are also present in lung tissue in high amounts [3]. All these proteins, in different ratios, build the lung structures such as bronchi, bronchioles, alveoli, and blood vessels (Table S1). Several ECM proteins are involved in the formation of a metastatic niche in pulmonary metastasis of primary breast cancer, and they accompany cancer progression. Among them, collagens, fibronectin, tenascin C, versican, periostin and others, are present. Collagens, implicated in modulation of cancer cell activities and fate, and other lung proteins are degraded by serine proteases and various metalloproteases (MMPs). An immunochemical study assessed the expression of elastin and MMPs-2, -4, and -9 in a murine model of pulmonary breast cancer metastasis, that showed the degradation of elastin is associated with an increased expression of these MMPs in lungs in the phases of micro- and macro-metastasis [4]. Gradual remodeling of ECM and its high dynamics do not induce compositional changes of ECM components, but they rather cause re-distribution of individual proteins, their cross-linking and spatial arrangement within the tissue. Unfortunately, the formation of new structures in fibers constrains specific staining of proteins in light and electron microscopy [3]. This issue requires searching for new label-free tools sensitive to molecular structures of proteins and showing their distribution in complex ECM-cancer interactions in the lung. 

One of the rapid, label-free and non-destructive methods useful for the examination of the composition and structures of molecules in tissues is Fourier Transform Infrared spectroscopic imaging (FTIR). FTIR spectroscopy is a technique successfully applied for the analysis of secondary structures of polypeptides and proteins, including purified proteins as well as those in biological samples [5,6,7,8]. A number of FTIR spectroscopic reports concerning investigations of collagen and elastin structures in biological samples were mainly focused on tissues with dominant contribution of these proteins to tissue composition. For instance, Camacho and co-workers investigated collagen type I and II as well as proteoglycans in bovine articular cartilage and observed differences in the localization of amide I and II bands specific for these proteins [8]. Whilst Petibois and others distinguished collagen type I from type IV after deconvolution of amide bands in FTIR spectra of ECM in skeletal muscle [9]. The detection of internal elastic lamina and smooth muscle cells surrounded by elastin and collagen by micro-ATR–FTIR imaging (Attenuated Total Reflection) has been shown for atherosclerotic lesions in rabbits [10]. The analysis of the amide I band in the spectra of the lesions indicated disordered conformation of proteins altered then due to a nitration effect [11]. In turn, Chrabaszcz et al. used large-area FTIR scanning of lung cross-sections to assess an ECM remodeling degree in lung structures at early and late phases of pulmonary metastasis of breast carcinoma and suggested that the amide III region of fibrous proteins is the most sensitive marker to track an effect of metastasis on lung parenchyma [12]. 

FTIR polarization contrast imaging (PCI) could be a relevant tool for probing linear anisotropy of fibrous structures forming extracellular media in tissues [13]. Polarized infrared light promotes vibrational excitation of functional groups only when the dipole change is aligned with the polarization of the incident field [14,15]. A few reports used this physical property to determine the relationship between mechanical damage of proteins and aging in tendon and skin due to altered orientation or cleavage of protein fibers. Parallel (p-) and perpendicular (s-) polarized FTIR spectra of undamaged tendon, mainly composed of collagen type I, showed significant differences in intensities of amide I and II bands [14]. This observation was used to identify regions of mechanically damaged mammalian tendon in which the axial alignment of the fibrils was lost. Polarized FTIR spectra of a rat tail tendon were also analyzed for the cutaneous chronologic aging in human skin. In this case, FTIR-PCI imaging indicated that fibers of collagen type I become parallel to the skin surface in aged skin dermis [16]. 

In this work we propose the combination of standard and polarized contrast FTIR spectroscopic imaging for the determination of protein alternations in the lung alongside the development of pulmonary breast cancer metastasis in an orthotopic mouse model. We carried out long-term observations to compare changes in the lung parenchyma at the pre-metastatic stage (week 2) (leukocyte infiltration is mainly recognised in conventional hematoxylin and eosin (H&E) staining without visible changes in lung morphology), an early micro-metastasis (week 3) (colocalization of inflammation single neoplastic cells or their small clusters) up to an advanced phase (week 5) (solid metastases of various sizes are present in lung parenchyma). Since we showed that standard FITR imaging could be successfully applied to detect single cancer cells [17], secondary tumors [18], and assess alternations of lung structures [12], we applied PCI imaging to advance current capabilities for the detection of early metastasis and its effect on a chemism of ECM.

## 2. Results

**Figure 1:**
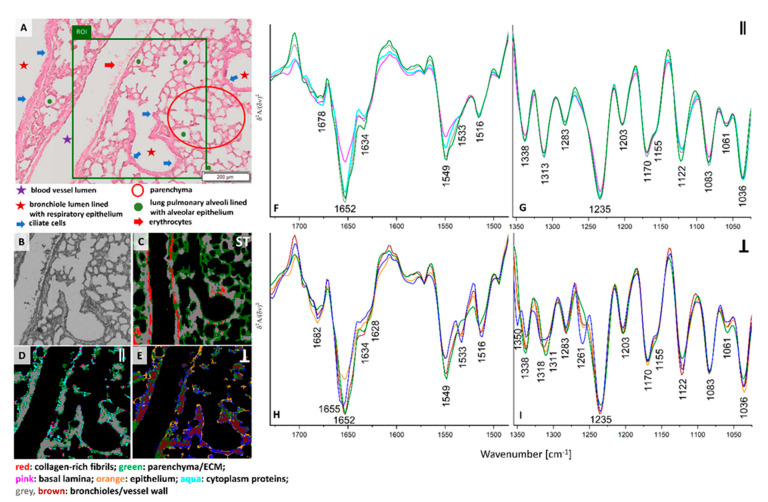
(**A**) A H&E micro-photography showing cellular phenotypes of lung parenchyma observed in healthy control and ROI (green area) imaged spectroscopically (magnification: 20×); (**B**) a white-field image of the ROI (magnification: 15×); false-colour UHCA maps for IR images recorded by using non-polarized FTIR imaging (**C**), 0° (**D**) and 90° (**E**) polarization contrast imaging. Mean second derivative FTIR spectra extracted from UHCA maps for 0° (**F**,**G**) and 90° (**H**,**I**) polarized IR light. Mean spectra of non-polarized FTIR imaging are collected in Figure S3E,F. The colors of classes correspond to the colors of spectra.

**Figure 2:**
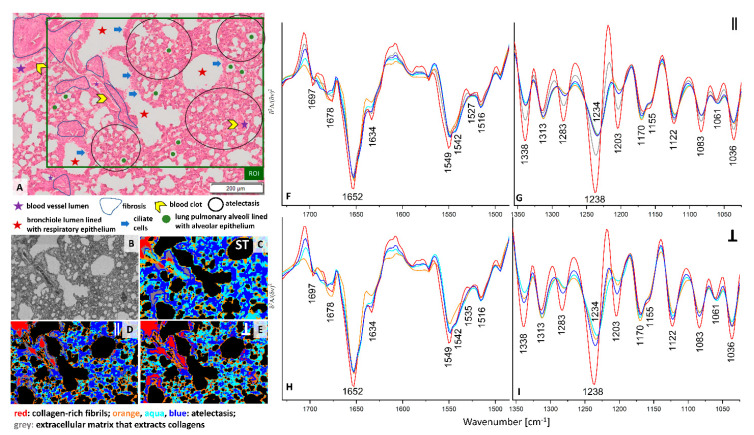
(**A**) A H&E micro-photography (20×) showing cellular phenotypes of lung parenchyma in the pre-metastatic phase (week 2) and ROI (green area) imaged spectroscopically (magnification: 20×); (**B**) a white-field image of the ROI (magnification: 15×); false-colour UHCA maps for IR images recorded by using non-polarized FTIR imaging (**C**), 0° (**D**) and 90° (**E**) polarization contrast imaging. Mean second derivative FTIR spectra extracted from UHCA maps for 0° (**F**,**G**) and 90° (**H**,**I**) polarized IR. Mean spectra of non-polarized FTIR imaging are collected in Figure S3G,H. The colors of classes correspond to the colors of spectra.

**Figure 3:**
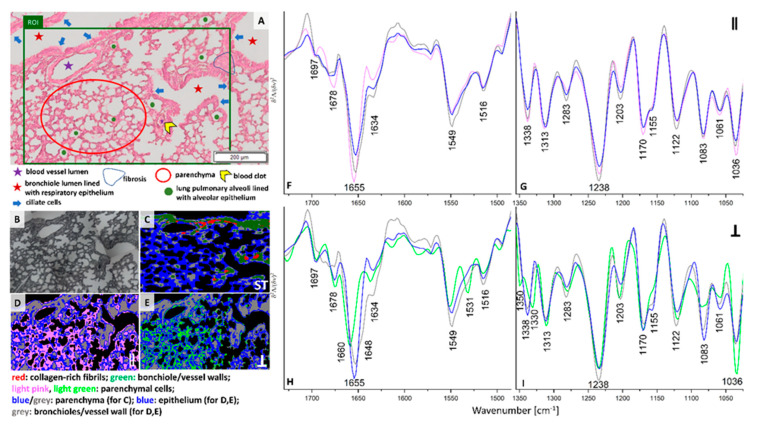
(**A**) A H&E micro-photography showing cellular phenotypes of lung parenchyma observed in the micro-metastasis phase (week 3) and ROI (green area) imaged spectroscopically (magnification: 20×); (**B**) a white-field image of the ROI (magnification: 15×); false-colour UHCA maps for IR images recorded by using non-polarized FTIR imaging (**C**), 0° (**D**) and 90° (**E**) polarization contrast imaging. Mean second derivative FTIR spectra extracted from UHCA maps for 0° (**F**,**G**) and 90° (**H**,**I**) polarized IR light. Mean spectra of non-polarized FTIR imaging are collected in Figure S3I,J. The colors of classes correspond to the colors of the spectra.

**Figure 4:**
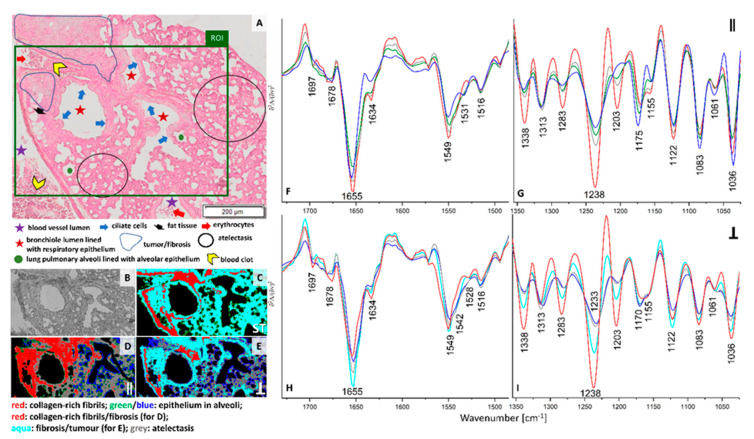
(**A**) A H&E micro-photography (20×) showing cellular phenotypes of lung parenchyma in in the macro-metastatic phase (week 5) and ROI (green area) imaged spectroscopically (magnification: 20×); (**B**) a white-field image of the ROI (magnification: 15×); false-colour UHCA maps for IR images recorded by using non-polarized FTIR imaging (**C**), 0° (**D**) and 90° (**E**) polarization contrast imaging. Mean second derivative FTIR spectra extracted from UHCA maps for 0° (**F**,**G**) and 90° (**H**,**I**) polarized IR. Mean spectra of non-polarized FTIR imaging are collected in Figure S3K,L. The colors of classes correspond to the colors of spectra.

To investigate time-dependent changes in ECM induced by cancer metastasis from breast to lung, critical for cancer development, we selected tissue cross-sections from the following phases of the disease development, the pre-metastatic (week 2), micro-metastatic (week 3), and macrometastasic (week 5) phases, and compared FTIR data with those obtained for healthy control (HC) (Figure 1, Figure 2, Figure 3 and Figure 4). FTIR images were collected by using irradiation of non-polarized, parallel (p-, i.e., 0°), and perpendicular (s-, i.e., 90°) polarized infrared radiation and we captured the same Regions of Interest (ROI) in all cases. Subsequently, H&E staining, used as a “standard” screening test for histopathological diagnosis, was performed on the same cross-sections to visualize lung morphology and to identify what lung structures exhibit variation in the protein composition gathered from the FTIR images. For the latter, Unsupervised Hierarchical Cluster Analysis (UHCA) was performed to reduce hyperspectral database and reveal spectral differences. Firstly, FTIR images constructed for ratios of amide I, II, and III bands show the overall changes in structure and orientation of proteins (Figure S1) and may reflect an increase in the rate of cell changes, deterioration of cell function and genome instability. Amide I and II bands in the region of 1700–1500 cm^−1^ are assigned to stretches of the C=O group and bends of the N-H bond in the amide bonding, respectively, and highlight secondary conformations of proteins and their alternation. In turn, the amide III band (the skeletal C-N and C-C stretching vibration) has a very specific spectral motif for fibrous proteins, i.e., a triad of peaks in the 1300–1200 cm^−1^ region [7]. Standard and PCI IR images displayed in Figure S1 differ from each other for each case and these differences become more pronounced when cancer cells infiltrated the lung (weeks 3 and 5), i.e., an increased intensity of amide I and III bands, with respect to amide II band, is observed in some regions of the tissue cross-sections. Although ATR FTIR spectra of the most abundant lung proteins, collagens type I, III, IV and elastin, exhibit distinct spectral profiles in the entire region between 1700 and 900 cm^−1^, the complex biological matrix of the tissues can hide their features (Figure S2). 

H&E staining of the lung cross-section of healthy control exhibits the presence of various morphological structures typical for lungs such as longitudinal cross-section through a blood vessel, bronchi, and bronchioles. Bronchioles are sent with ciliated monolayer epithelium and contain bronchiolar cells (Clary) secreting proteins and glycosaminoglycans. A myofibroblasts, individual macrophages, mast cells and numerous blood vessels in contact with type I pneumocytes and type II pneumocytes are present in healthy control (Figure 1A). The ROI selected for FTIR imaging includes the pulmonary artery with erythrocytes, bronchioles and fine-lanced parenchyma with alveoli (Figure 1A,B). UHCA analysis of the conventional FTIR image differentiated three classes assigned mainly to bronchioles and walls of blood vessels together (grey class), pulmonary parenchyma with alveoli (green class), collagen-rich fibrils in the longitudinal cross-section of the venous vessel wall (red class) (Figure 1C and Figure S3). For the latter, the presence of collagens is confirmed by FTIR spectrum of neat collagens, in particular in the region below 1350 cm^−1^ (see Figures S2 and S3F). The non-polarized FTIR spectrum of the red class depicted an increased absorbance of β-sheet structures (amide I: 1697 and 1634 cm^−1^) and bands at 1338, 1283, 1238, and 1203 cm^−1^ attributed to the δ(CH_2_), δ(CH_3_), ν(C–N), and δ(N–H) absorptions of collagens, respectively [19]. A broad feature at 1155 cm^−1^ suggest the dominance of collagen type I and IV in this class what is congruent with the protein composition of the vessel wall (Table S1). The remaining structures of the healthy lungs, i.e., walls of vessel and bronchioles and parenchyma assigned to the grey and green classes, differ between themselves mainly in the region of amide I and II bands and by the intensity of the carbohydrate band at 1036 cm^−1^ higher in alveoli than in vessels and bronchioles (Figure S3E,F). On the contrary to the collagen-rich class, these structures exhibit a high level of α-helices in their proteins (amide I: 1652 cm^−1^), and a low contribution of β-sheet structures. A significant decrease in intensity of the collagen amide III bands and the presence of the 1170 cm^−1^ band indicate the co-existence of collagens and elastin, (Figures S2C,D and S3F).

Surprisingly, the collagen fibrils observed earlier are not visualized in healthy control by irradiation of neither parallel nor perpendicular polarized IR light (see Figure 1D–I). The cluster maps and their mean FTIR spectra clearly show substantial differences in distribution of classes and chemical features (Figure 1C–I). Hierarchical analysis of the 0⁰ polarization FTIR image mainly indicates the presence of walls of blood vessels and bronchioles clustered together into the grey class (Figure 1D). Interestingly, the other classes show the distribution of very minute cells such as the epithelium in alveoli (green class) mixed with single pixels assigned to basal lamina (pink class) and cytoplasm proteins (aqua class). The spectral profiles of all classes mainly vary in intensities of the amide I and II bands while the band positions exhibit the presence of collagens and elastin (Figure 1F,G). In addition, we observe in Figure S1 a higher amide I: amide II ratio for the middle part of the walls of vessels and bronchioles than for the other tissue fragment that can indicate protein fibers oriented parallelly along the wall. Furthermore, conventional and 0-degree polarized FTIR imaging classify parenchyma, walls of blood vessels and bronchioles together in contrast to polarized IR light at 90° that reveal different distribution of spectral features (see Figure 1E,H,I). Walls of blood vessels and bronchioles (grey class in Figure 1C,D) are now segregated into bronchiolar and vascular walls (brown class, Figure 1E) surrounded by endoplasmic reticulum and its cytoplasm (blue class) and accompanied by ECM and collagen fraction (green class). Additionally, the epithelium that lines the alveoli and bronchioles were distinguished as an orange class. Perpendicular polarization of infrared radiation exhibits pronounced spectral differences, i.e., a shift of the amide I band from 1652 cm^−1^ to 1655 cm^−1^ is observed for the blue and orange traces and the increased intensity of the 1533 cm^−1^ band appeared in all classes, except the green one (Figure 1H). The latter also shows the lack of the new amide III band at 1261 cm^−1^ which is pronounced for the blue class (Figure 1I). According to a report of Zhang and co-workers, the bands at 1655, 1533, and 1261 cm^−1^, present in the IR spectrum of the blue class localized around vessel and bronchiole walls, originate from random scaffolds and cross-linking of collagens on contrary to aligned protein morphologies in the core of the walls (brown class) [20]. Other tissue structures revealed by perpendicular IR light very likely possess a blend of proteins of different orientations.

H&E stain of the ROI selected in the cross-section of the pre-metastatic stage of pulmonary cancerogenesis (week 2) shows the presence of a longitudinal cross-section through a blood vessel and bronchioles surrounded by fibrosis (Figure 2A). In this metastatic phase, a fine-lance and healthy lung parenchyma is mostly replaced by atelectasis, a condition where the lung does not fill up with enough air, which is manifested by the thickening of the parenchyma and narrowing of the alveoli lumen as a result of growing inflammatory infiltrate. Clustering of standard and PCI FTIR images distinguished four classes with similar distribution through the tissue cross-section (Figure 2C–E). All red classes represent the collagen-rich regions with the spectral profile described above (Figure 2F–I and Figure S3G,H). UHCA analysis of the conventional IR image differentiated two classes in the region labelled as fibrosis, i.e., the red and grey classes. The grey areas surround the blood vessel and the neighboring bronchiole lumen while the red class is assigned to histological features showing less cell accumulation with pronounced ECM that extracts collagens (Figure 2A,C). The FTIR spectra of the two classes shows an intensity increase in the α-helical amide I band (1652 cm^−1^) for fibrosis surrounding the venous blood vessel and bronchiole and a decrease in absorbances specific for amide III bands of collagens (grey trace) compared to the solid-like fibrotic transformation of parenchyma (red) (Figure S3G,H). Surprisingly, the spectral features of the red classes observed in the blood vessel of healthy control and fibrosis in the pre-metastatic phase are very similar, so we hypothesize that we accidentally found dysfunctional changes in the large vessel of the 6-week old control mouse or some collagen scaffolds in the large vessels resemble the spectral signature of fibrosis. 

Interestingly, illumination of the lung cross-section with parallel polarized IR light (0°) indicates that the collagen-rich IR class (red areas) covers most of the area occupied by fibrosis while the grey class is present at the edges of this lung tissue transformation (Figure 2C,F,G). The grey areas differ from the red areas class only by lowering the intensity of the amide III region, similarly to conventional FTIR spectra. In turn the 90° PCI image shows a high content of collagens across the entire region of fibrosis (Figure 2E,H,I). The localization of the collagens in the three IR images agree with the physiology of fibrotic scarring in that these proteins are produced by fibroblasts leading to accumulation of ECM components due to inflammation typically observed before invasion of cancer cells [21].The evidence for the structural and spatial distribution of collagen fibers are changes in the regions of amide I and III bands observed in mean IR spectra of the red classes. Cluster analysis of conventional and PCI IR images show that atelectasis is spectrally characterised by three classes (blue, aqua, and orange) where the spectra mainly differ by intensities of numerous bands in the region of amide I and II bands. The appearance of the amide II signal at 1542 cm^−1^ is specific for this deformation of lung parenchyma compared to a control (Figure 1 and Figure 2).

Colonization of single cancer cells or their clusters in lungs appear around three weeks of the breast cancer progression in the orthotropic murine model [22]. Our previous studies on early micro-metastasis phase in this model showed that single cancer cells or their clusters occupy 0.002–0.12% per imaged tissue area (ca. 5.5 mm^2^). This makes their identification difficult for H&E histopathology [12,17]. H&E staining of the chosen ROI exhibits the presence of blood micro-vessels, bronchi, and bronchioles surrounded by parenchyma (Figure 3A). Lung parenchyma is open-work and formed by thin-walled pulmonary alveoli and this fragment of the tissue does not show signs of atelectasis, inflammation or fibrosis observed in other regions of this lung lobe (data not shown). So, this ROI, in principle, exhibits histologically features of healthy lung. Conventional FTIR cluster this ROI into four classes attributed to collagens-rich cells (red) localized in the bronchiole and vessel walls (green), and parenchyma (blue and grey) (Figure 3C and Figure S3I,J). Interestingly, both polarizations of IR irradiation images result in similar distribution of classes, but different than in the conventional IR image (Figure 3C–E). The grey classes include the bronchioles and vessel walls while the blue ones are classified as epithelium. The spectral profiles of grey and blue classes share similarities in both parallel and perpendicular polarization. In addition, the spectral characteristics of the IR polarized parenchymal cells in the range of 0° and 90° differ significantly. Therefore, they were grouped into two classes of pink and light green (Figure 3F–I). The spectrum for 0° polarized light is similar to the corresponding spectrum of parenchyma in healthy control, except a 3 cm^−1^ up-shift of the main amide I band (pink and green traces in Figure 2 and Figure 3F,G). Whereas the perpendicularly polarized IR spectrum of parenchyma shows a further shift of the amide I band from 1655 to 1660 cm^−1^ and the appearance of the amide II and III bands at 1533, 1350, and 1330 cm^−1^. These changes in protein conformations are accompanied by a strong decrease in intensity of signals at 1155 and 1082 cm^−1^ attributed to the carbohydrate moieties in collagens and glycoproteins (light green trace in Figure 3H,I). This indicates modifications in protein structure from triple-helices and helices into turns and β-sheets due to degradation, cross-linking and re-orientation of collagen fibers induced by cancer metastasis. During the cancer progression collagen degradation is enhanced by pro-inflammatory M1 macrophages recruited into the tissue by the altered activity of cancer cells [23]. 

Solid secondary tumor in the lung is fully developed on week 5 after inoculation of breast cancer cells. An enlarged micro-photography of H&E staining of the investigated cross-section is displayed in Figure S4. A marked area of tumor is overlapped with fibrotic tissue since it is impossible to distinguish these structures separately; the tumor arises from fibrosis without a clear anatomic border. FTIR imaging included this fragment as well as cross-sections of blood vessel with blood clot, bronchi, and bronchioles (Figure 4A and Figure S4). In this macro-metastatic phase, normal lung parenchyma is mostly replaced by atelectasis with visible inflammation and fibrous tissue. Inflammatory infiltration is histologically recognised by an increased density of the tissue, the lack of air spaces in the lung, and the presence of pink cytoplasm in parenchymal cells due to the production of pro-inflammatory proteins.

Clustering of IR spectra obtained in conventional and PCI imaging exhibit distinct distribution of classes (Figure 4C–E). In each UHCA map, we observe the red class assigned to the collagen-rich structures that fully overlap with the tumor and fibrosis, but only when the sample was irradiated with 0° polarized IR. The latter suggests the co-existence of fibrous proteins with the same orientation in these structures. We also observe that clustering of this image results from intensity variation in the spectral region below 1350 cm^−1^, in particular for 1180–1000 cm^−1^ bands that revealed vibrations of carbohydrate moieties. These changes could be related with re-building of ECM associated with cross-linking of fibrous proteins or their decomposition and through alternation of the content of the sugar groups. For non-polarized and perpendicular infrared light, a high level of collagens is mainly detected in the longitudinal cross-section of vessel wall, around basement membrane of bronchiole and at the edge of the fibrotic tissue. FTIR spectra gathered with the conventional imaging approach merges pixels from the area assigned to atelectasis, fibrosis and tumor (aqua class in Figure 4C) while 90° polarized FTIR spectra discriminated these tissue structures into aqua (fibrosis and tumor) and grey classes (atelectasis) (Figure 4E). Green and blue classes in the three UHCA maps show the presence of epithelium in alveoli. FTIR spectra of the perpendicular polarization and non-polarized IR light are clustered into classes due to changes in intensity in the entire spectral region (Figure 4H,I and Figure S3K,L). 90° polarized PCI segregated fibrosis (aqua) from atelectasis (grey) based on the increased intensities of amide I and III bands indicating that fibrosis possesses more α-helical conformations aligned perpendicularly to the tissue surface with some contribution of collagens than in deformed parenchyma. 

## 3. Discussion

The detailed studies of the tumor micro-environment may increase the knowledge about potential mechanisms and development and progression of the metastatic disease [24]. The lungs are often the first sites of metastasis in nearly one quarter of metastatic breast cancer patients. Thus, the lungs were chosen as the organ of metastasis to be studied in our research. Taking into account that all mice developed lung metastasis upon orthotopic implantation of a metastatic subline of 4T1 cells (breast cancer cell line derived from the mammary gland tissue of a mouse BALB/c strain), this cell line was used. Furthermore, unlike most of the transgenic mouse models of breast cancer metastasis, 4T1 tumor cells also metastasize to the bone, thereby, resembling human breast cancer metastasis [25]. The two-polarization FTIR spectroscopic approach was applied to the lung cross-sections to reveal for the first time the orientational ordering of proteins upon the development of pulmonary metastasis of breast cancer. Their distribution was complemented by the detailed histological examination. The protein composition is very complex in the lung tissue, but the main morphological structures are built of collagens type I, III, IV, and elastin. Their dominant content is easily detected by FTIR spectroscopy. Imaging with non-polarized infrared light, widely used in spectral histopathology, indicated the presence of collagen-rich spots localized primarily in the walls of vessels and bronchioles, as well as in fibrotic tissue. FTIR spectra acquired in that way and then clustered by UHCA showed similar signals where the intensity altered upon metastasis progression. On the other hand, polarized contrast imaging recognised tissue structures of the lungs and showed deformations in proteins scaffolds induced by inflammatory infiltration, fibrosis, and tumor growth. This effect can be recognised by the changes in band intensities, band shifts and the appearance of new bands, e.g., at ca. 1530 cm^−1^ in the lung parenchyma in the micro-metastatic phase and 1260 cm^−1^ in cytoplasm within bronchioles in control. Both bands were observed in IR image polarized IR at 90°. The former could be a marker of cellular modification in the lung in the phase when breast cancer cells started to cross the vessel barrier.

Table 1 summarized the changes in the ratio of integrated intensities of amide I, II, and III bands in UHCA classes assigned to morphological structures of lungs that are considered to reflect fibril orientations along the tissue surface. The formation of the pre-metastatic niche preparing the lung environment for settling of metastatic cancer cells through remodeling of ECM was clearly indicated by the amide I to amide II ratio for parenchyma and epithelium. This ratio gradually increased along with metastasis development in the spectra of parenchyma whereas it dropped down in epithelium of the pre-metastatic phase only when the polarization was at 0°. Whilst the perpendicular polarization of IR light was used, the amide I: amide II ratio increased already up to week 3 and then it decreased again in fully remodeled alveoli to a value similar for week 2. 

The changing ratio of amide III and I bands also exhibited anisotropy under the polarization conditions for parenchyma and epithelium. This ratio is almost constant during metastasis, except the micro-metastatic phase. The enhancement of the collagen triple bands can suggest random alignment collagens when they were secreted due to signaling between lung fibroblasts. 

Infrared light absorption is reliant on the angular orientation of transition moments of individual molecular group vibrations; therefore, it can be used to determine the conformational states of molecules with respect to electrical field vectors of polarized IR radiation [26]. For anisotropic samples the absorption of polarized light varies and depends on its direction, creates an effect of linear dichroism represented by dichroic ratio (D) is defined as D=A_‖(0⁰)_/A_Ʇ(90⁰)_ where A_‖_ and A_Ʇ_ represent absorbances for parallel and perpendicular radiation, respectively. Values of this ratio give the insights about the dipole moment orientation distribution with regards to the orientation axis as well as might be used for Herman’s orientation function (f) also called in-plane orientation function [27]. The latter describes the degree or extent of orientation of the chain axes (polymers, fibers) relative to some other axis of interest and is expressed by the equation below:

(1) $$f=\frac{D-1}{D+2}\frac{2}{3{cos}^{2}\alpha -1\;}$$

where α is an angle between the transition dipole moment and main molecular axis [26]. Calculated values of Herman’s orientation function vary in the range of −0.5 to 1 corresponding to low- and high-ordered structures. The isotropic character of sample is indicative by zero. 

**Figure 5:**
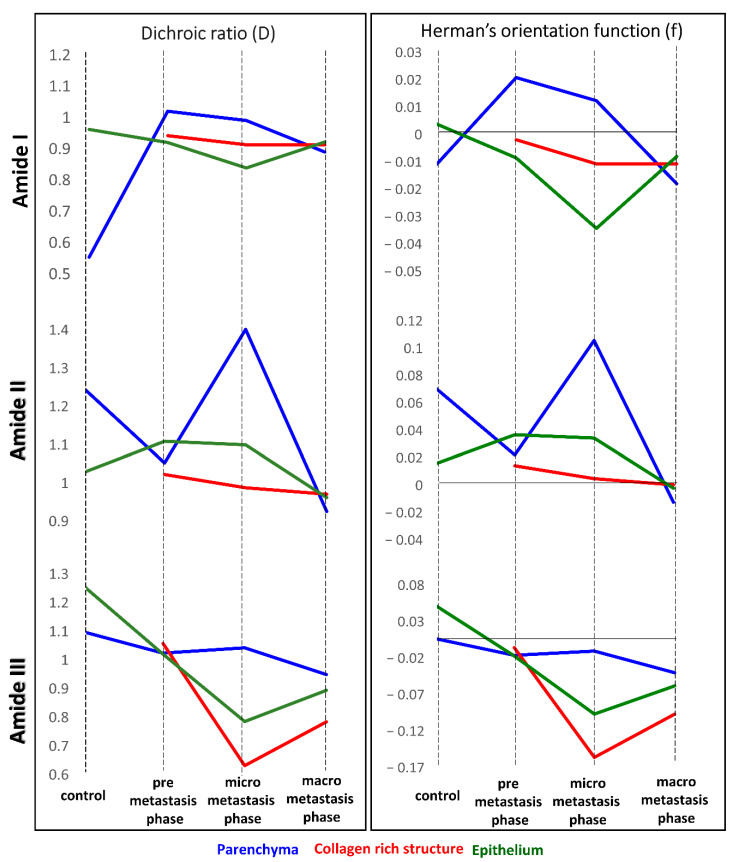
The graph shows the values of D and f and their changes depending on the phase of metastasis from the Table 2.

Based on integral intensities of amide I, II and III, we calculated dichroic ratio (D) and the Herman’s orientation function (f) (Table 2) and summarised their as graphs in Figure 5. These functions are effective to understand the tendency of proteins orientation and the subtle changes which induce trend of their behaviour, triggered by invading cancer cells and to define relationship with the cancer metastasis. It is known that the local fibers alignment promotes cancer cells interactions with ECM [28]. 

The fibrotic tissue (**^●^**), present for each discussed amide, exhibits orientation changes towards disordered structures of proteins, which are the most prominent for the macro-metastatic phase, where these fibers surround the cancerous lesions without obvious specific alignment. In contrast, a degree of disorganization is lower than in vessel and bronchiole walls fraction with high collagenous contribution (▫), present in micro-metastasis.

Parameters calculated for amide III bands regard the collagen fibers present in the lung tissue. Collagens in parenchyma and epithelium form random alignment due to ECM remodeling, however this process occurs earlier in epithelium and is noticeable in the micro-metastatic phase. The highest value of the factor f for the amide III band is determined for fibrotic tissue in the pre-metastatic phase suggesting ordering of collagens fibers. This is congruent with previous observations showing that collagens with filament-like structures display the alignment towards cancer cell invasive directions [28]. Therefore, when affected, reorient towards parallel alignment to promote their migration inside ECM.

Values of Herman’s function for the amide I and II bands suggest the contribution of proteins other than collagens (Table 2). A close to zero value found for healthy epithelium indicates the isotropic orientation of its proteins which becomes disordered with the metastasis development. 

This tendency is not observed when the developed tumors are present. Other proteins assigned to amide II become less oriented in pre metastasis phase and undergo former reorientation in micro- and become disoriented in macro-metastasis. It might result from the activity of epithelial cells mediating the actinomyosin contractility involved in fibers reorganization, when the spheroid cancer cells cause traction forces and provoke anisotropic orientation [28]. In parenchyma, ordering of polypeptide chains in proteins occurs in the micro-metastasis phase when the activity of matrix metalloproteinases is pronounced (Table 2). When the lung is occupied by large metastatic foci, all proteins undergo mis-organization. 

ECM proteins are extremely important for the formation of metastasis and colonization in a distant metastatic site [1]. Currently, numerous studies have been conducted on different functions of proteins representing the tumor’s immediate environment. For instance, Oskarsson and co-workers have proved that ECM proteins like Tenascin-C (TNC) and Versican (VCAN) play a critical role during the earliest stage of breast cancer metastasis to the lungs [29]. In a Malanchi report, periostin (POSTN) was identified as a component of the ECM and is expressed by fibroblasts in healthy tissue and by stroma of the primary tumor in the breast. Infiltrating cancer cells induce stromal POSTN expression in the secondary target organ—the lungs—to initiate colonization [30]. Another study showed that ECM components may provide a favorable environment for disseminated cancer cells to interact with other cells. Among them vascular cell adhesion molecule-1 (VCAM-1) is abnormally expressed in breast cancer cells and binds to α4β1 integrin, which further interacts with fibronectin. Pulmonary parenchyma containing collagen and elastin fibers acts as a favorable environment for homing of breast cancer expressing VCAM-1 [31]. The investigation of the pre-metastatic niche and tumor microenvironment is fundamental to understand mechanisms of the cancer development. We believe that application of Fourier Transform Infrared polarization contrast imaging will contribute to improved understanding of the process of proteins degradation in lungs upon metastasis from breast cancer.

## 4. Materials and Methods

### 4.1. Sample Preparation and Histological Analysis

Lung tissue samples were possessed from inbred mouse strains BALB/cAnNCrl (control) (*n* = 1) and BALB/cAnNCrl (*n* = 1) within 2, 3, and 5 weeks after orthotopic inoculation with viable 4T1 tumor cells of metastatic breast cancer. Isolated lungs were washed in saline and fixed with 4% formalin buffered solution for 48 h. Paraffin-embedded cross-sections of tissues were cut from the middle of the isolated left lung. Then, 7 μm thick cross-sections were prepared using a paraffin method on an Accu-Cut^®^ SRM™ 200 Rotary microtome mounted on CaF_2_ windows and dewaxed before FTIR imaging. After the collection of FTIR spectroscopic images, the same regions of interest (ROI) in a cross-section were imaged with the use of PCI in light polarization of 0 and 90°. After all, FTIR imaging measurements, cross-sections were stained with haematoxylin and eosin (H&E) for the histopathological examination. The examination and photographic documentation of dyed slides were performed using an Olympus BX53F white-light microscopic equipped with a DP74 digital camera. All investigations presented in this work conformed to the Guide for Care and Use of Laboratory Animals published by the US National Institutes of Health. A local animal research committee approved the experimental procedures used in the present study (permit no. LKE140/2013).

Protein standards were selected based on the lung tissue ECM composition and literature [32,33]. Here we studied human collagen type I (CC050, Sigma Aldrich, St. Louis, MO, USA), human collagen type III (CC054, Sigma Aldrich), human collagen type IV (CC076, Sigma Aldrich) and elastin from mouse lung (E6402, Sigma Aldrich). All collagens were solutions of 1 mg/mL purified protein liquids containing 0.5 M acetic acid, except elastin that was lyophilized powder due to its insolubility.

### 4.2. Conventional and Polarized Contrast FTIR Spectroscopic Imaging and Data Analysis

For conventional FTIR spectroscopic imaging of lung cross-sections, we used a combination of an Agilent 670-IR FTIR spectrometer and a 620-IR microscope working in rapid mode, which allows for the collection of 16,384 spectra from an area of circa 495,616 μm^2^ within 90 s (Santa Clara, CA, USA). A focal plane array (FPA) detector cooled with liquid nitrogen was coupled with this equipment. The detector consisted of a matrix of 16,384 pixels, arranged in a 128 × 128 grid format. The collection of IR images was performed in transmission mode. FTIR spectroscopic imaging used a 15× Cassegrain objective and condenser optics with numerical aperture (NA) of 0.62 and a projected FPA pixel size of 5.5 μm × 5.5 μm, giving a measured area of ca. 704 μm × 704 μm. All FTIR spectra were recorded by co-adding of 64 scans and in the range of 3800 to 900 cm^−1^ with a spectral resolution of 4 cm^−1^.

Polarized FTIR spectroscopic images were collected using a Hyperion 3000 FTIR microscope coupled with Tensor 27 spectrometer (Bruker Optics, Bullerica, MA, USA) at 15× magnification (NA = 0.4) using a rotatable polarizer (Bruker Optics, Bullerica, MA, USA) placed at the polarizer holder of the microscope for experiments in transmission mode. A 64 × 64 FPA was used that simultaneously measured an area of 170 × 170 μm^2^. Imaging was combined with mapping to obtain FTIR images of the size of the ROIs. The FTIR spectra were recorded at a spectral resolution of 4 cm^−1^ with 128 co-added scans.

Protein standards were measured as dried films (collagens) or lyophilizates (elastin) with the use of Agilent 670-IR spectrometer equipped with an ATR diamond crystal (Santa Clara, CA, USA). Spectra were recorded by co-adding of 256 scans in the range from 4000 to 570 cm^−1^ with a spectral resolution of 4 cm^−1^. 

Pre-processing and chemometric analysis of the acquired FTIR images were performed using CytoSpec (ver. 2.00.01) [34], MatLab (R2015a, Natick, MA, USA), and Origin 9.1 (ver. 2018b, OriginLab, Northampton, MA, USA) software. Firstly, a quality test was employed to introduce a threshold level and to eliminate signals with an absorbance lower than 0.2 and greater than 1.2. This operation was performed in the region between 1620 and 1680 cm^−1^. To remove spectral noise, we executed principal component analysis (PCA) based noise reduction with 15 principal components (PCs). Resonant Mie scattering EMSC correction using seven PCs was performed on all spectra [35]. Next, second derivative IR spectra were calculated with 13 smoothing points according to the Savitzky–Golay protocol. All spectra were then vector normalized in the 914 to 1770 cm^−1^ region to account for the differentiation in the sample thickness. Unsupervised hierarchical cluster analysis (UHCA) was executed in the spectral region of 1770–970 cm^−1^ using second derivative FTIR spectra. Spectral distances were computed as D-values and the individual clusters were extracted according to Ward’s algorithm. The narrowed spectral region excluding the range above 1800 cm^−1^ was chosen for UHCA analysis due to the contribution of the trace content of paraffin to absorbances of the stretching C-H vibrations.

The five ATR-corrected FTIR spectra of pure protein were averaged and then second derivative spectra were computed with 13 smoothing points according to the Savitzky–Golay protocol. Next, these spectra were vector normalized in the 4000–900 cm^−1^ region. All steps were performed with the use of the OPUS 7.0 program (Bruker Optics, Bullerica, MA, USA, Version 7.2.139.1294) and presented as graphs with the use of the Origin 9.1 software.

## 5. Conclusions

Cancer progression is associated with numerous changes in the morphology of the affected organs. These changes are based on biochemical processes caused by the activity of constantly spreading cancer cells. Although classical histopathology followed by biochemical methods is the gold standard in clinical and experimental oncology and have high sensitivity and specificity in detecting markers, the method is somewhat subjective when there is a need to discriminate specific abnormalities. Infrared imaging can overcome this and combine morphological aspects and biochemical compositional information in tissue sections.

In the presented study we showed that Fourier Transform Infrared polarization contrast imaging recognizes proteins degradation in lungs upon metastasis from breast cancer. This paper shows the potential for the use of conventional and polarized contrast FTIR spectroscopic imaging, a “spectral histopathology”, to characterize biochemical changes of the metastatic target. This determines phenotypes of tissue structures and deliver a novel spectroscopic marker panel for the recognition of the metastatic environment. Thus, these features can allow for a better understanding of processes like proteins degradation occurring in diseased tissues during cancer progression. The conventional and polarized contrast FTIR spectroscopic imaging could provide hints what staining methods are appropriate for specific detection of the metastatic processes. The detailed studies of the tumor micro-environment can broaden our knowledge about potential mechanisms and development and progression of the metastatic disease. The results presented in this work substantially extend the currently existing knowledge of orientational ordering of proteins upon progression of pulmonary metastasis of breast cancer. Our research shows that determination of phenotypes of tissue structures and indicated a high potential for the discovery of new markers, using spectroscopic methods, for the recognition of the metastatic environment. Our work demonstrates the possibilities of FTIR spectroscopy and spectroscopic imaging as rapidly developing physico-chemical methods that are increasingly used in biomedical research and clinical diagnostics.

## Figures and Tables

**Table 1:** Correlation of calculated ratios for each stage of cancer progression, standard and polarized FTIR spectroscopic imaging for different morphological elements occurred in cross-sections. Integration range Amide I (1620–1680 cm^−1^)/Amide II (1560–1480 cm^−1^); Amide III (1350–1185 cm^−1^)/Amide I (1620–1680 cm^−1^).

TISSUE STRUCTURE	FTIR	CONTROL	PRE- METASTATIC PHASE (Week 2)	MICRO- METASTATIC PHASE (Week 3)	MACRO- METASTATIC PHASE (Week 5)
**Amide I/Amide II**					
**Parenchyma**	ST	2.17	2.30	2.26	2.36
	0°	2.37	2.37	2.41	2.47
	90°	2.21	2.40	3.10	2.47
**Collagen-rich structure**	ST	2.31 ⁺	2.40 ^●^	2.36 ⁺	2.39 *
	0°	-	2.33 ^●^	2.34 ▫	2.45 *
	90°	-	2.40 ^●^	2.80 ▫	2.48 *
**Epithelium**	ST	-	2.18	2.26	2.30
	0°	2.40	2.32	2.41	2.43
	90°	2.32	2.44	2.80	2.42
**Amide III/Amide I**					
**Parenchyma**	ST	0.19	0.15	0.18	0.16
	0°	0.15	0.13	0.19	0.13
	90°	0.17	0.13	0.19	0.13
**Collagen-rich structure**	ST	0.20 ⁺	0.18 ^●^	0.19 ⁺	0.17 *
	0°	-	0.15 ^●^	0.14 ▫	0.15 *
	90°	-	0.14 ^●^	0.18 ▫	0.18 *
**Epithelium**	ST	-	0.19	0.18	0.18
	0°	0.16	0.16	0.15	0.13
	90°	0.14	0.16	0.18	0.14

**⁺** collagens fibers in longitudinal cross-section of vessel and bronchiole wall; **^●^** fibrotic tissue; ▫ high collagenous contribution in vessel and bronchiole wall; * tumor with fibrosis.

**Table 2:** Correlation of calculated dichroic ratio (D) and orientation function (f) for different morphological elements occurred in cross-sections according to each stage of cancer progression for polarized FTIR spectroscopic imaging.

TISSUE STRUCTURE	FTIR	CONTROL	PRE- METASTATIC PHASE(Week 2)	MICRO- METASTATIC PHASE (Week 3)	MACRO- METASTATIC PHASE(Week 5)
**Amide I-C = O (1620–1680 cm^−1^)**					
Parenchyma	D	0.95583	1.07578	1.0432	0.93135
	f	−0.01494	0.02464	0.01419	−0.02342
Collagen-rich structure	D	-	0.98945 ^●^	0.95724 ▫	0.95734 *
	f	-	−0.00353 ^●^	−0.01446 ▫	−0.014425 *
Epithelium	D	1.01139	0.96525	0.87455	0.96699
	f	0.00378	−0.01172	−0.04364	−0.01112
**Amide II-C-N, -N-H (1560–1480 cm^−1^)**					
Parenchyma	D	1.20903	1.04020	1.34128	0.93093
	f	0.06514	0.01322	0.10214	−0.02356
Collagen-rich structure	D	-	1.01434 ^●^	0.98440 ▫	0.97001 *
	f	-	0.00476 ^●^	−0.00523 ▫	−0.01007 *
Epithelium	D	1.01894	1.08911	1.08113	0.96233
	f	0.00627	0.02885	0.02633	−0.01272
**Amide III (1350–1185 cm^−1^)**					
Parenchyma	D	1.08269	1.00911	1.02695	0.93659
	f	0.02682	0.00303	0.00890	−0.02159
Collagen-rich structure	D	-	1.04158 ^●^	0.62933 ▫	0.7773 *
	f	-	0.01367 ^●^	−0.14098 ▫	−0.08017 *
Epithelium	D	1.24185	1.0060	0.77804	0.88357
	f	0.07460	0.0020	−0.07989	−0.04038

**^●^** fibrotic tissue; ▫ high collagenous contribution in vessel/bronchiole wall; * tumor with fibrosis.

## Data Availability

The data presented in this study are available on request from the corresponding author. The data are not publicly available due to their massive file size.

## Supplementary Materials

The following are available online at https://www.mdpi.com/2072-6694/13/2/162/s1, Figure S1. Integral intensity images for standard and PCI FTIR imaging based on ratio: I. amide I (1620–1680 cm−1) to amide II (1560–1480 cm−1) bands, II. fibrous proteins (amide III, 1350–1185 cm−1) to amide I (1620–1680 cm−1) bands. The band ratios differentiate image contrast based on lung tissue proteins due to ECM remodeling induced by breast cancer metastasis. Figure S2. Second derivative ATR FTIR spectra of pure collagen I (black), III (red), IV (blue), and elastin (green) in the regions of 900–1700 cm−1 (A), amide I and II bands (B), amide III band (C) and stretching and deformation vibrations of the C-O, C-C and C-OH groups specific for sugar moieties (D). Figure S3. False-colour UHCA maps (A-D) of standard FTIR imaging of lung cross-sections derived from control, pre- (week 2), micro- (week 3), and macro-metastatic (week 5) phases of pulmonary metastasis of breast carcinoma. The colour of the classes corresponds to the colors mean second derivative FTIR spectra (E-L). Figure S4. Enlarged H&E micro-photography of the lung cross-section from week 5 with a marked region of solid tumor (left). The green ROI (right) was investigated with FTIR imaging (Figure 4).

## References

[1] Jin L., Han B., Siegel E., Cui Y., Giuliano A., Cui X. (2018). Breast cancer lung metastasis: Molecular biology and therapeutic implications. Cancer Biol. Ther..

[2] Dunsmore S.E., Rannels D.E. (1996). Extracellular matrix biology in the lung. Am. J. Physiol..

[3] Green E.M., Mansfield J.C., Bell J.S., Winlove C.P. (2014). The structure and micromechanics of elastic tissue. Interface Focus.

[4] Smeda M., Kieronska A., Adamski M.G., Proniewski B., Sternak M., Mohaissen T., Przyborowski K., Derszniak K., Kaczor D., Stojak M. (2018). Nitric oxide deficiency and endothelial-mesenchymal transition of pulmonary endothelium in the progression of 4T1 metastatic breast cancer in mice. Breast Cancer Res..

[5] Staniszewska E., Malek K., Baranska M. (2014). Rapid approach to analyze biochemical variation in rat organs by ATR FTIR spectroscopy. Spectrochim. Acta Part A Mol. Biomol. Spectrosc..

[6] Surewicz W.K., Mantsch H.H. (1988). New insight into protein secondary structure from resolution-enhanced infrared spectra. Biochim. Biophys. Acta.

[7] Riaz T., Zeeshan R., Zarif F., Ilyas K., Muhammad N., Safi S.Z., Rahim A., Rizvi S.A.A., Rehman I.U. (2018). FTIR analysis of natural and synthetic collagen. Appl. Spectrosc. Rev..

[8] Camacho N.P., West P., Torzilli P.A., Mendelsohn R. (2001). FTIR microscopic imaging of collagen and proteoglycan in bovine cartilage. Biopolymers.

[9] Petibois C., Gouspillou G., Wehbe K., Delage J.P., Déléris G. (2006). Analysis of type i and IV collagens by FT-IR spectroscopy and imaging for a molecular investigation of skeletal muscle connective tissue. Anal. Bioanal. Chem..

[10] Palombo F., Cremers S.G., Weinberg P.D., Kazarian S.G. (2009). Application of Fourier transform infrared spectroscopic imaging to the study of effects of age and dietary L-arginine on aortic lesion composition in cholesterol-fed rabbits. J. R. Soc. Interface.

[11] Palombo F., Shen H., Benguigui L.E.S., Kazarian S.G., Upmacis R.K. (2009). Micro ATR-FTIR spectroscopic imaging of atherosclerosis: An investigation of the contribution of inducible nitric oxide synthase to lesion composition in ApoE-null mice. Analyst.

[12] Chrabaszcz K., Kaminska K., Augustyniak K., Kujdowicz M., Smeda M., Jasztal A., Stojak M., Marzec K.M., Malek K. (2020). Tracking Extracellular Matrix Remodeling in Lungs Induced by Breast Cancer Metastasis. Fourier Transform Infrared Spectroscopic Studies. Molecules.

[13] Tuchin V.V. (2016). Polarized light interaction with tissues. J. Biomed. Opt..

[14] Wiens R., Findlay C.R., Baldwin S.G., Kreplak L., Lee J.M., Veres S.P., Gough K.M. (2016). High spatial resolution (1.1 μm and 20 nm) FTIR polarization contrast imaging reveals pre-rupture disorder in damaged tendon. Faraday Discuss..

[15] Hikima Y., Morikawa J., Kazarian S.G. (2019). Analysis of molecular orientation in polymeric spherulite using polarized micro attenuated total reflection Fourier transform infrared (ATR-FTIR) spectroscopic imaging. Anal. Chim. Acta.

[16] Nguyen T., Eklouh-Molinier C., Sebiskveradze D., Feru J., Bouland N., Terryn C., Manfait M., Brassart-Pasco S., Piot O. (2014). Changes of skin collagen orientation associated with chronological aging as probed by polarized- FTIR micro-imaging. Biomed. Spectrosc. Imaging.

[17] Chrabaszcz K., Jasztal A., Smęda M., Zieliński B., Blat A., Diem M., Chlopicki S., Malek K., Marzec K.M. (2018). Label-free FTIR spectroscopy detects and visualizes the early stage of pulmonary micrometastasis seeded from breast carcinoma. Biochim. Biophys. Acta Mol. Basis Dis..

[18] Augustyniak K., Chrabaszcz K., Jasztal A., Smeda M. (2019). High and ultra-high definition of infrared spectral histopathology gives an insight into chemical environment of lung metastases in breast cancer. J. Biophotonics.

[19] Belbachir K., Noreen R., Gouspillou G., Petibois C. (2009). Collagen types analysis and differentiation by FTIR spectroscopy. Anal. Bioanal. Chem..

[20] Zhang M., Li Z., Jiang P., Lin T., Li X., Sun D. (2017). Characterization and cell response of electrospun Rana chensinensis skin collagen/poly(l-lactide) scaffolds with different fiber orientations. J. Appl. Polym. Sci..

[21] Sapalidis K., Sardeli C., Pavlidis E., Koimtzis G., Koulouris C., Michalopoulos N., Mantalovas S., Tsiouda T., Passos I., Kosmidis C. (2019). Scar tissue to lung cancer; pathways and treatment. J. Cancer.

[22] Smeda M., Przyborowski K., Proniewski B., Zakrzewska A., Kaczor D., Stojak M., Buczek E., Nieckarz Z., Zoladz J.A., Wietrzyk J. (2017). Breast cancer pulmonary metastasis is increased in mice undertaking spontaneous physical training in the running wheel; a call for revising beneficial effects of exercise on cancer progression. Am. J. Cancer Res..

[23] Xu S., Xu H., Wang W., Li S., Li H., Li T., Zhang W., Yu X., Liu L. (2019). The role of collagen in cancer: From bench to bedside. J. Transl. Med..

[24] Bubnov R., Polivka J., Zubor P., Konieczka K., Golubnitschaja O. (2017). “Pre-metastatic niches” in breast cancer: Are they created by or prior to the tumour onset? “Flammer Syndrome” relevance to address the question. EPMA J..

[25] Berman A.T., Thukral A.D., Hwang W.T., Solin L.J., Vapiwala N. (2013). Incidence and patterns of distant metastases for patients with early-stage breast cancer after breast conservation treatment. Clin. Breast Cancer.

[26] Bolterauer C., Heller H. (1996). Calculation of IR dichroic values and order parameters from molecular dynamics simulations and their application to structure determination of lipid bilayers. Eur. Biophys. J..

[27] Koziol P., Liberda D., Kwiatek W.M., Wrobel T.P. (2020). Macromolecular orientation in biological tissues using a four-polarization method in FT-IR imaging. Anal. Chem..

[28] Han W., Chen S., Yuan W., Fan Q., Tian J., Wang X., Chen L., Zhang X., Wei W., Liu R. (2016). Oriented collagen fibers direct tumor cell intravasation. Proc. Natl. Acad. Sci. USA.

[29] Oskarsson T., Acharyya S., Zhang X.H.-F., Vanharanta S., Tavazoie S.F., Morris P.G., Downey R.J., Manova-Todorova K., Brogi E., Massagué J. (2011). Breast cancer cells produce tenascin C as a metastatic niche component to colonize the lungs. Nat. Med..

[30] Malanchi I., Santamaria-Martínez A., Susanto E., Peng H., Lehr H.-A., Delaloye J.-F., Huelsken J. (2011). Interactions between cancer stem cells and their niche govern metastatic colonization. Nature.

[31] Sharma R., Sharma R., Khaket T.P., Dutta C., Chakraborty B., Mukherjee T.K. (2017). Breast cancer metastasis: Putative therapeutic role of vascular cell adhesion molecule-1. Cell. Oncol..

[32] Zhou Y., Horowitz J.C., Naba A., Ambalavanan N., Atabai K., Balestrini J., Bitterman P.B., Corley R.A., Ding B.S., Engler A.J. (2018). Extracellular matrix in lung development, homeostasis and disease. Matrix Biol..

[33] Frantz C., Stewart K.M., Weaver V.M. (2010). The extracellular matrix at a glance. J. Cell Sci..

[34] Lasch P. Cytospec: Software for Hyperspectral Imaging. http://www.cytospec.com.

[35] Bassan P., Kohler A., Martens H., Lee J., Byrne H.J., Dumas P., Gazi E., Brown M., Clarke N., Gardner P. (2010). Resonant Mie Scattering (RMieS) correction of infrared spectra from highly scattering biological samples. Analyst.

