# Supplementary material for: Fourier Transform Infrared Polarization Contrast Imaging Recognizes Proteins Degradation in Lungs upon Metastasis from Breast Cancer
Source: Cancers (Basel). 2021 Jan 6;13(2):162. doi: 10.3390/cancers13020162 (PMC7825053; doi:10.3390/cancers13020162)
Supplement: Supplementary file 1 [file cancers-13-00162-s001.pdf]

cancers

Supplementary materials: Fourier Transform Infrared Polarization Contrast Imaging Recognizes Proteins Degradation in Lungs upon Metastasis from Breast Cancer

Karolina Chrabaszcz, Katarzyna Kaminska, Cai Li Song, Junko Morikawa, Monika Kujdowicz, Ewelina Michalczyk, Marta Smeda, Marta Stojak, Agnieszka Jasztal, Sergei G. Kazarian and Kamilla Malek

Table S1. A summary of main morphological structures in lungs and their dominant building proteins.

	Bronchioles and bronchi	*Alveoli*	*Venous vessels wall*	*Arterial vessels wall*	*Lung parenchyma*	*ECM*
Dominant proteins	collagen I, III, IV<br>laminin<br>elastin<br>fibronectin	collagen I, III, IV<br>laminin<br>elastin	collagen I, IV<br>laminin<br>fibronectin	collagen I, IV<br>elastin<br>laminin<br>fibronectin	collagen I, IV<br>elastin<br>laminin<br>fibronectin	collagen I, III, IV, V, XI<br>laminin<br>fibronectin<br>transcenin

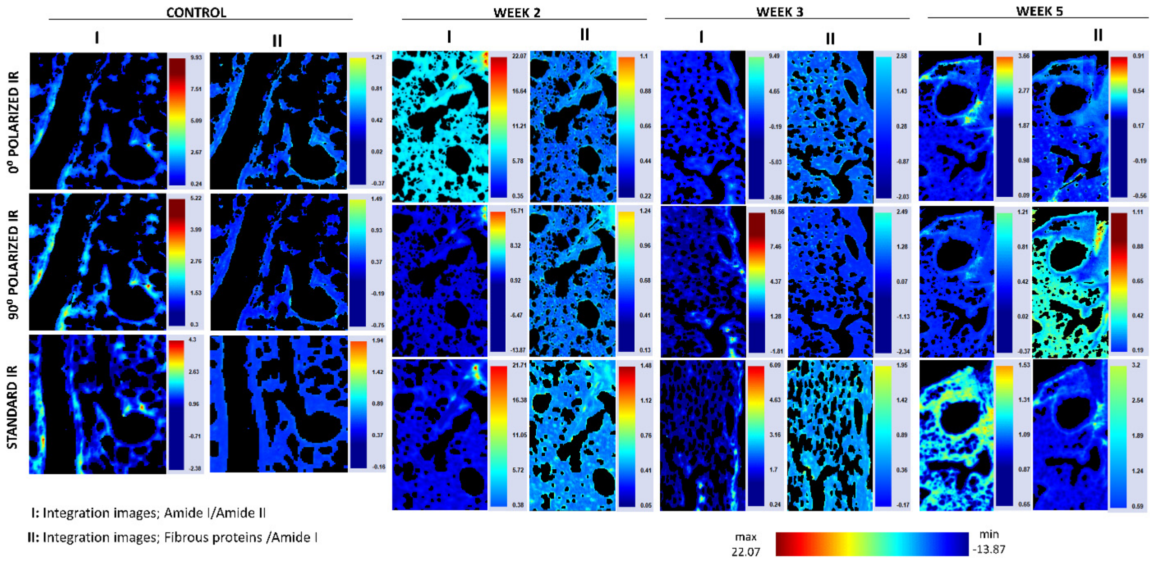

Figure S1. Integral intensity images for standard and PCI FTIR imaging based on ratio:

I. amide I (1680–1620cm^{-1}) to amide II (1560–1480 cm^{-1}) bands,

II. fibrous proteins (amide III, 1350–1185 cm^{-1}) to amide I (1680–1620 cm^{-1}) bands.

The band ratios differentiate image contrast based on lung tissue proteins due to extracellular matrix remodelling induced by breast cancer metastasis.

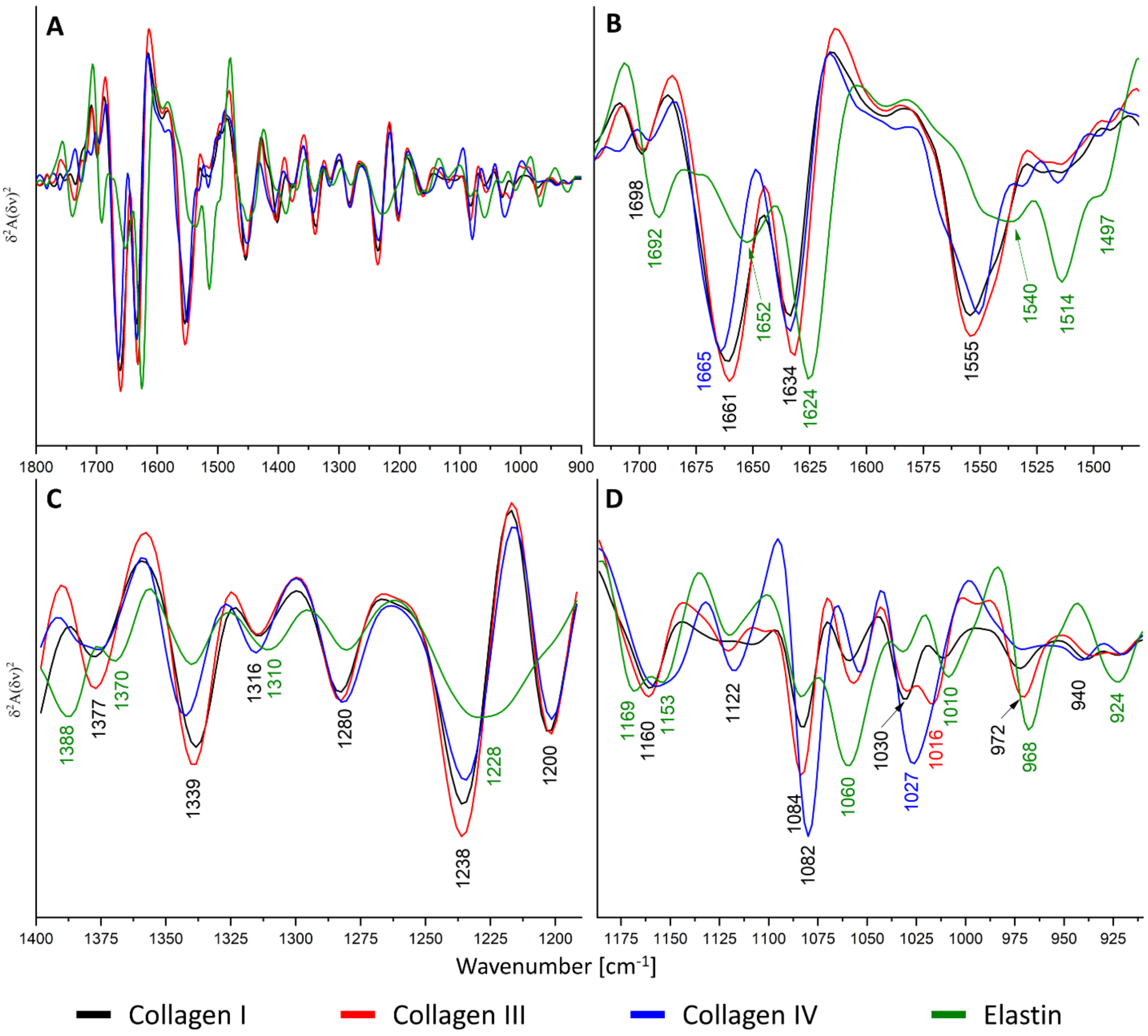

Figure S2. Second derivative ATR FTIR spectra of pure collagen I (black), III (red), IV (blue) and elastin (green) in the regions of 1700 - 900 cm^{-1} (**A**), amide I and II bands (**B**), amide III band (**C**) and stretching and deformation vibrations of the C-O, C-C and C-OH groups specific for sugar moieties (**D**).

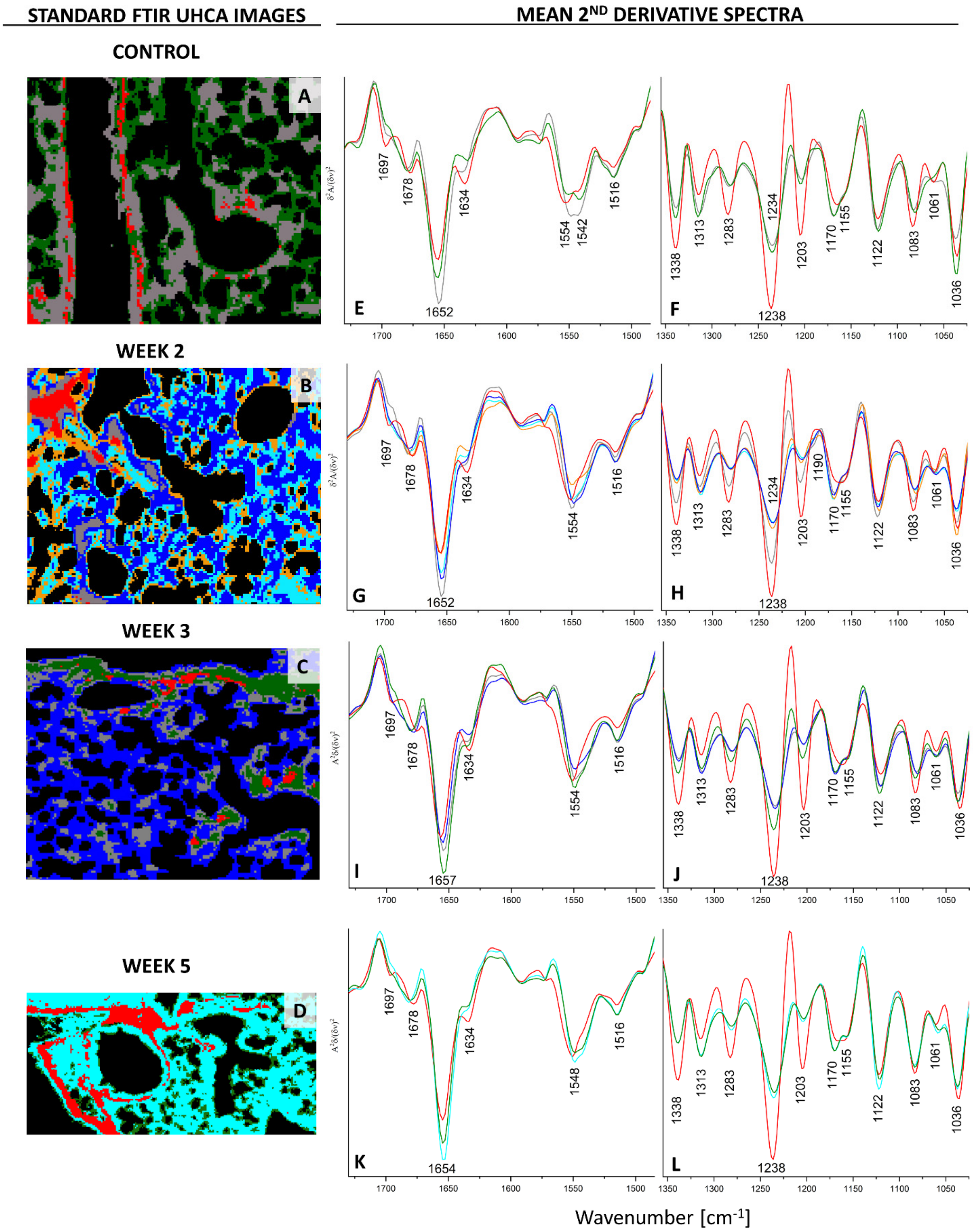

Figure S3. False-colour UHCA maps (**A–D**) of standard FTIR imaging of lung cross-sections derived from control, pre- (week 2), micro- (week 3), and macro-metastatic (week 5) phases of pulmonary metastasis of breast carcinoma. The colour of the classes corresponds to the colours of the mean second derivative FTIR spectra (**E–L**).

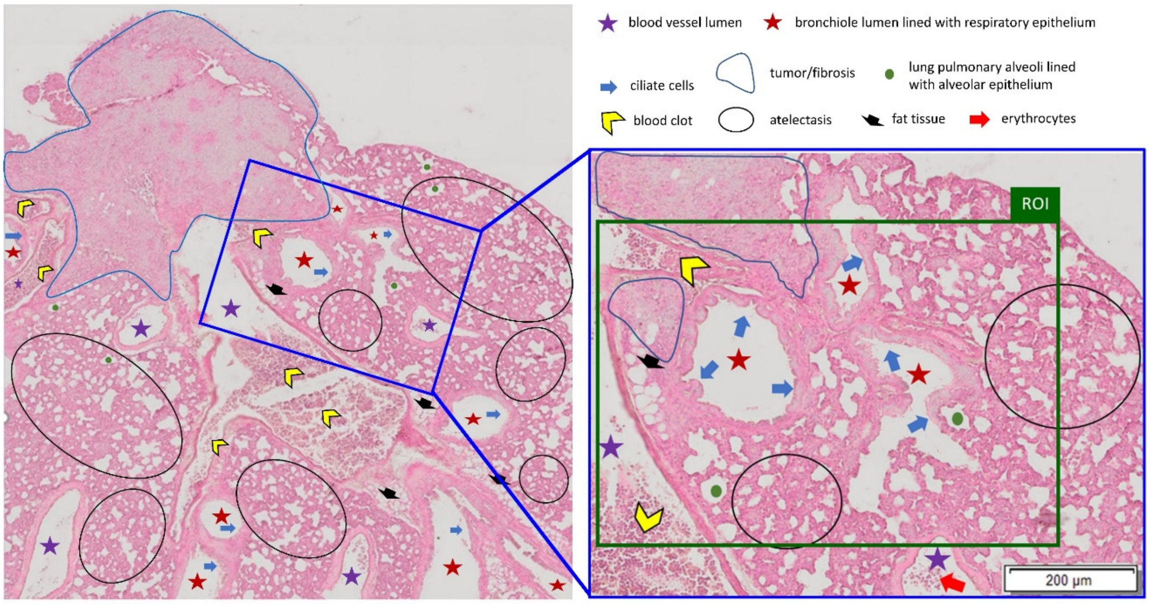

Figure S4. Enlarged H&E microphotography of the lung cross-section from week 5 with a marked region of solid tumour (left). The green ROI (right) was investigated with FTIR imaging, Fig. 4.
